# Supported Bimetallic AuPd Nanoparticles as a Catalyst for the Selective Hydrogenation of Nitroarenes

**DOI:** 10.3390/nano8090690

**Published:** 2018-09-05

**Authors:** Ruiyang Qu, Margherita Macino, Sarwat Iqbal, Xiang Gao, Qian He, Graham John Hutchings, Meenakshisundaram Sankar

**Affiliations:** 1 Cardiff Catalysis Institute, School of Chemistry, Cardiff University, Cardiff CF10 3AT, UK; qury@zju.edu.cn (R.Q.); macinom@cardiff.ac.uk (M.M.); iqbals13@cardiff.ac.uk (S.I.); heq6@cardiff.ac.uk (Q.H.); hutch@cardiff.ac.uk (G.J.H.); 2 State Key Laboratory of Clean Energy Utilization, College of Energy Engineering, Zhejiang University, Hangzhou 310027, China

**Keywords:** bimetallic nanoparticles, solvent free hydrogenation, selective hydrogenation of nitrobenzene and selective hydrogenation of chloronitrobenzene

## Abstract

The solvent-free selective hydrogenation of nitrobenzene was carried out using a supported AuPd nanoparticles catalyst, prepared by the modified impregnation method (M_Im_), as efficient catalyst >99% yield of aniline (AN) was obtained after 15 h at 90 °C, 3 bar H_2_ that can be used without any further purification or separation, therefore reducing cost and energy input. Supported AuPd nanoparticles catalyst, prepared by M_Im_, was found to be active and stable even after four recycle experiments, whereas the same catalyst prepared by S_Im_ was deactivated during the recycle experiments. The most effective catalyst was tested for the chemoselective hydrogenation of 4-chloronitrobenzene (CNB) to 4-chloroaniline (CAN). The activation energy of CNB to CAN was found to be 25 kJ mol^−1^, while that of CNB to AN was found to be 31 kJ mol^−1^. Based on this, the yield of CAN was maximized (92%) by the lowering the reaction temperature to 25 °C.

## 1. Introduction

**Scheme 1:**
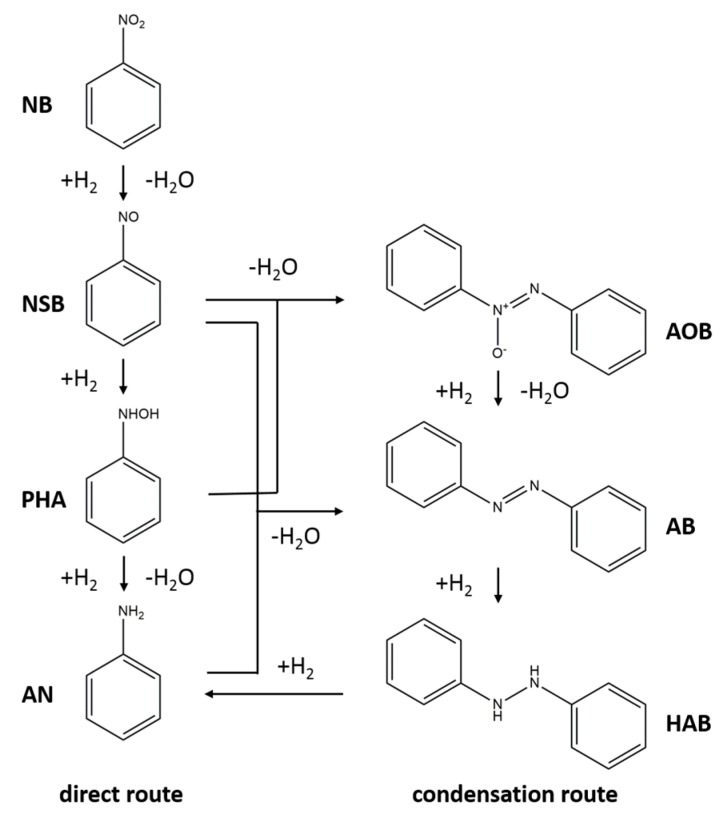
Widely accepted reaction mechanism of the hydrogenation of nitrobenzene to aniline initially proposed by Haber [7].

Aniline (AN) is an industrially very important intermediate for the production of dyes, agricultural chemicals, pharmaceuticals, polymers, etc. [1,2,3]. About 85% of aniline is produced via the catalytic hydrogenation of nitrobenzene (NB) using gaseous H_2_ under either liquid or gas phase conditions, as it is environmentally benign, and water is the only by-product in this reaction [4]. Moreover nitration of aromatic compounds is a very well established and optimized technology, which makes nitroarenes a readily available feedstock for the production of bulk and/or fine chemicals [5,6]. The Haber reaction mechanism, as shown in Scheme 1, was the generally accepted reaction mechanism for the nitrobenzene hydrogenation for a long time [7]. Aniline can be formed via two routes. In the direct route, NB is reduced to nitrosobenzene (NSB), then to N-phenylhydroxylamine (PHA), and finally, to form AN. The condensation route involves the formation of azoxybenzene (AOB) from the condensation of NSB and PHA. AOB is then reduced to azobenzene (AB) and hydrazobenzene (HAB) as intermediates. HAB is finally hydrogenated to AN. In this route, condensation of NSB and AN to form AB may occur as well.

Solvents, such as ethanol [1,8,9,10,11,12,13], methanol [14], and 2-propanol [15] have been used to facilitate heat and mass transfer during liquid-phase reaction conditions. However, after the end of the reaction, additional separation steps are always employed to separate the product, resulting in not only high cost and energy inputs, but also a higher process E-factor [16]. One way to circumvent this problem is to perform the reaction under solvent-free conditions, which is a green and atom-economical process. Sun and co-workers [4] developed an ultrafine Pt nanoparticles supported on a multi-walled carbon nanotube catalyst for the “solvent-free” hydrogenation of nitrobenzene; however, the target product AN was used as a solvent for this hydrogenation reaction. Recently, Huang and co-workers [17] reported monometallic Pd nanoclusters supported on an N-doped ordered mesoporous carbon catalyst for the solvent-free hydrogenation of NB. This monometallic catalyst has been reported to display good activity and selectivity for the reduction of various substituted nitro aromatics to their corresponding amines. However, the synthesis of this catalyst is complicated, which makes it challenging for potential industrial application.

Recently, several groups including ours, have reported supported Pd-based bimetallic nanoparticles as very active and selective catalysts compared to their monometallic analogues, for the direct synthesis of hydrogen peroxide from hydrogen and oxygen, selective oxidation, selective hydrodeoxygenation, and many more transformations [18,19,20,21,22]. Similar to monometallic catalysts, the synthesis strategy plays a crucial role in determining the activity, selectivity, and stability of supported bimetallic catalysts. We have developed a number of synthesis strategies to control the particle size, composition, size dependent composition, and nanostructure of supported AuPd nanoparticles, including wet impregnation, sol immobilization, modified impregnation, chemical vapour deposition, etc. [23,24]. The objective of this work is to develop very active, selective, and stable supported bimetallic AuPd nanoparticulate catalysts for the solvent-free hydrogenation of nitrobenzene.

Chloroaniline (CAN), an intermediate for the production of pesticides, drugs, and dyes, is produced by the reduction of chloronitrobenzene, which in turn is produced by the nitration of chlorobenzene. However, the hydrogenation of chloronitrobenzene (CNB) to chloroaniline is a challenging task, as the weak carbon–halogen bond is highly susceptible to cleavage, resulting in the formation of AN, and thus lowering the selectivity of CAN [25,26]. Fine tuning of Pd- [27,28], Pt- [26,29], and Ni-based [30] catalysts through nanostructure modification, selective poisoning, and other strategies, have been reported. However, for a given catalyst, the optimization of reaction conditions to increase the selectivity of CAN has not been reported yet.

Based on these two themes, the aim of the present work is to develop an efficient and robust catalyst for the solvent-free hydrogenation of nitrobenzene, and to investigate this system in detail through kinetic studies and activation energy calculation. The best catalyst will be utilized for the chemoselective hydrogenation of CNB to CAN, and the reaction conditions will be optimized to increase the selectivity of CAN.

## 2. Experimental

### 2.1. Catalyst Preparation

The catalysts were prepared by modified impregnation, conventional impregnation and sol-immobilization methods, as reported by our group previously elsewhere [31,32]. The total metal loading used here was 1 wt %. For the bimetallic AuPd catalyst, the weight percentages of Au and Pd were both 0.5 wt %.

Modified Impregnation (M_Im_): A gold precursor solution was prepared by dissolving HAuCl_4_·3H_2_O (Sigma Aldrich Company Ltd., Gillingham, UK) in deionized water and palladium precursor solution was prepared by dissolving PdCl_2_ (Sigma Aldrich Company Ltd., Gillingham, UK) in a 0.58 M aqueous HCl solution. The desired amounts of gold solution and/or palladium solution were placed into a 50 mL round-bottom flask. The total volume of the solution was filled up to 16 mL with deionized water. The flask then was put into an oil bath sitting on a hotplate with a magnetic stirrer. The solution was stirred at 1000 rpm vigorously, and it was heated from room temperature to 60 °C for 10 min. The support (1.98 g, TiO_2_ P25 (Evonik Degussa, Essen, Germany), carbon (Cabot Vulcan XC72R, Cabot Carbon Ltd., Sully, UK), or MgO (BDH, Poole, UK), was then slowly added to the solution over a period of 10 min. The slurry was stirred for another 15 min, followed by heating from 60 to 95 °C, and was held for an additional 16 h with continuous stirring to evaporate the water. The solid powder was subsequently transferred to a mortar and ground thoroughly three times to form a uniform mixture. The sample was stored and denoted as a “dried only” catalyst. Before the reaction, a portion of the dried only sample (400 mg) was reduced using 5% H_2_/Ar at 400 °C for 4 h in a furnace. The sample was finally denoted as a M_Im_ catalyst.

Conventional Impregnation (C_Im_): In this preparation procedure, PdCl_2_ was dissolved in deionized water instead of aqueous HCl solution. Other procedures were the same as for the modified impregnation method.

Sol Immobilization (S_Im_): Requisite amounts of gold solution, palladium solution and freshly prepared polyvinylalcohol (PVA, Sigma Aldrich Company Ltd., Gillingham, UK) 1 wt % aqueous solution (Molecular Weight = 10,000, 80% hydrolyzed, PVA/(Au+Pd) (*w*/*w*) = 1:3) were dissolved in deionized water (400 mL) in a large beaker with vigorous stirring. A freshly prepared NaBH_4_ solution (0.1 M, NaBH_4_/(Au+Pd) (mol/mol) = 5) was then quickly added to form a dark brown solution. After continuous stirring for 30 min, the support material (TiO_2_) was added into the beaker. Then, one drop of concentrated H_2_SO_4_ was also added under vigorous stirring. After 2 h the slurry was filtered, and the catalyst was washed with deionized water (2 L) and dried in an oven at 120 °C overnight.

Scanning transmission electron microscopy characterization of 1% AuPd/TiO_2_ catalyst prepared by C_Im_, S_Im_, and M_Im_, was carried out using a JEOL JEM-2200FS (JEOL Ltd., Tokyo, Japan) aberration-corrected electron microscope at Lehigh University.

### 2.2. Catalytic Testing

Solvent-free hydrogenation of nitrobenzene: Nitrobenzene (78 mmol, ≥99.0%, Sigma Aldrich Company Ltd., Gillingham, UK) and catalyst (0.1 g) were placed in a Colaver^®^ glass reactor (Colaver, Milan, Italy). The molar ratio of the substrate/metal was 10,778. Before starting the reaction, the reactor was purged with N_2_ first, followed by pressurizing with H_2_ five times. The reactor was then charged with 3 bar H_2_ under isobaric conditions, and heated to 90 °C in an oil bath with a magnetic stirrer bar to start the reaction. After a selected reaction time, the reactor was cooled down to <5 °C using an ice bath, followed by depressurization. The reaction mixture was then taken out, centrifuged to remove the solid catalyst, and filtered before being injected into a gas-chromatographer (Varian 450-GC, Agilent, Santa Clara, CA, USA) equipped with a flame ionization detector (FID), a methanizer and a CP-SiL5CB column (50 m, 0.33 mm, Thermo Fisher Scientific, Newport, UK), in which He was used as the carrier gas. A calibration plot was utilized for the quantification of substrate and products. For the solvent free reaction, an equal amount of reaction mixture (0.5 mL) and external standard *o*-xylene (0.5 mL) were added and accurately weighted in a gas chromatographer (GC)vial. Whereas for reactions in present of solvent, the reaction mixture (1 mL) and the external standard *o*-xylene (0.1 mL) were added to a GC vial prior to analysis. The reactant and products were separated based on the boiling point (AN = 184.1 °C, NB = 210.9 °C, CAN = 232 °C, CNB = 242 °C). The program temperature increased from 70 °C to 250 °C at 10 °C/min to allow for separation and detection of the peaks. Identification and assignment of the chromatogram peaks was initially performed by Gas chromatography coupled with mass spectrometry Gas Chromatography coupled with Mass Spectrometer (Waters GCT Premier Mass Spectrometer, Waters Corporation, Milford, MA, USA) analysis.

Reusability test: Two batches of catalysts were used for the reusability test of the catalysts: batch A0: standard amount of catalyst (0.1 g); batch B0: an excess amount of catalyst (1.0 g). The reaction condition and operation procedure were identical to the solvent-free hydrogenation reaction. After 6 h reaction, the reaction mixture in batch A0 was analyzed by GC. The result was regarded as the activity for the fresh catalyst. The catalyst in batch B0 was taken out, stirred, and washed with acetone six times, followed by drying in an oven for 30 min at 110 °C. The standard amount of catalyst in batch B0 (0.1 g) was then taken as batch A1, and the rest was denoted as batch B1. Both batches were set up for reaction. The result of batch A1 was regarded as the activity for the first reuse. The catalyst in batch B1 was regenerated. The procedure was repeated up to the fourth reuse of the catalyst.

Kinetic study: For kinetic studies, ethanol (16 mL, ≥99.8%, Sigma Aldrich Company Ltd., Gillingham, UK) was used as the solvent for the hydrogenation reaction. Nitrobenzene (10 mmol) and catalyst (12.5 mg) were used for this test. The reaction temperature was selected as 40, 50, and 60 °C. The conversion was kept below 20%, to study the initial rate of the reaction. Other reaction conditions and operation procedures were the same with the solvent-free reaction.

Chemoselective hydrogenation of 4-chloronitrobenzene: Ethanol (16 mL) was used as the solvent for this hydrogenation reaction. 4-chloronitrobenzene (10 mmol, 99%, Sigma Aldrich Company Ltd., Gillingham, UK) and catalyst (12.5 mg) were used. Reaction temperature was selected as 20, 40, 50, and 60 °C. Other reaction conditions and operation procedures were the same as with the solvent-free reaction hydrogenation of nitrobenzene.

## 3. Results and Discussion

The solvent-free hydrogenation of nitrobenzene was performed using 1% AuPd/TiO_2_ (M_Im_) catalyst at different temperatures (30, 60, and 90 °C) (Table 1). Poor NB conversion and high AN selectivity was observed at 30 °C after a 2 h reaction. Increasing the reaction temperature, obviously increased the rate of NB conversion, while maintaining high AN selectivity. At 90 °C, the NB conversion was 24%, and AN selectivity was 98% after 2 h. Hence, 90 °C was selected as the standard reaction temperature for the further NB hydrogenation studies that were reported in this article.

In an effort to find the best metal, support, and synthesis method combination, we prepared different catalysts and tested them for the solvent-free hydrogenation of NB at 90 °C. We prepared monometallic 1% Au/TiO_2_ (M_Im_) and 1% Pd/TiO_2_ (M_Im_) catalysts, and their catalytic activities were compared with the bimetallic 1% AuPd/TiO_2_ (M_Im_) catalyst (Table 2). As expected, the bimetallic 1% AuPd/TiO_2_ (M_Im_) catalyst was found to be more active (54%) compared to the monometallic catalysts (3% and 41% for Au and Pd respectively) under identical reaction conditions. In previous reports published by our group, a similar phenomenon was observed also for other reactions such as selective oxidation [22,33], direct synthesis of H_2_O_2_ [34], and hydrogenation of levulinic acid to γ-valerolactone [20].

We checked the effect of the support. The AuPd supported on carbon showed only a 14% conversion of nitrobenzene and 76% selectivity towards aniline. When MgO was used as a support, the NB conversion was 36%, with 94% selectivity of aniline. Nevertheless, in both cases, the catalytic performances, including NB conversion and AN selectivity, were less than that of AuPd supported on TiO_2_. Thus, TiO_2_ was selected as the best support.

The synthesis strategy of the supported metal nanoparticles play a crucial role in determining the structural parameters, such as particle size, composition, and nanostructure, and hence their catalytic properties such as activity, selectivity, and stability [31]. As shown in Entries 6 and 7 in Table 2, the AuPd/TiO_2_ catalysts prepared by S_Im_ and C_Im_ showed similar NB conversions (39% and 38% respectively) and AN selectivities, while both were less than those of the M_Im_ catalyst (54%). From the data presented in Table 2, it is clear that 1% AuPd/TiO_2_ (M_Im_) is the best catalyst for the solvent-free hydrogenation of NB to AN.

**Figure 1:**
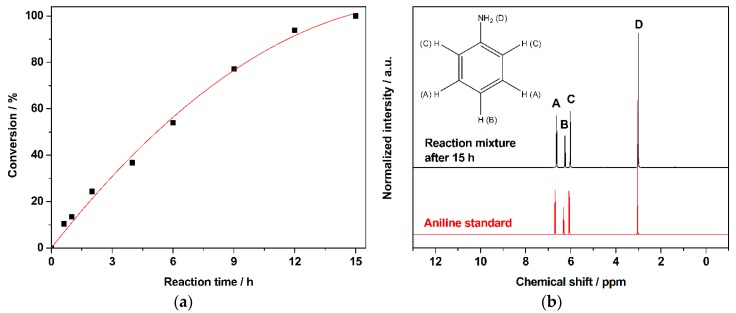
(**a**) Time-on-line plot for the solvent-free hydrogenation of nitrobenzene over 1% AuPd/TiO_2_ (M_Im_) catalyst. Reaction conditions: nitrobenzene: 78 mmol, catalyst: 100 mg, temperature: 90 °C, H_2_ pressure: 3 bar. (**b**) Comparison of the ^1^H-NMR spectra of the crude reaction mixture after 15 h without any purification and commercial aniline standard from Sigma Aldrich (>99.5% Purity).

Time on line evolution of products was studied using 1% AuPd/TiO_2_ (M_Im_) catalyst at 90 °C, and the result was shown in Figure 1a. After 15 h reaction, the entire NB was converted, and the GC yield of aniline was ca. 99% (GC did not show any other peak). To further confirm the formation and purity of aniline, we compared the ^1^H-NMR spectra of crude reaction mixture after 15 h of the reaction without any purification, with that of commercial aniline (≥99.5% purity from Sigma Aldrich Company Ltd., Gillingham, UK) as shown in Figure 1b. From this ^1^H-NMR spectra comparison it is clear that the crude reaction product is as pure as commercial AN standard, hence, the crude reaction mixture can be used for further application without any modification or purification.

**Figure 2:**
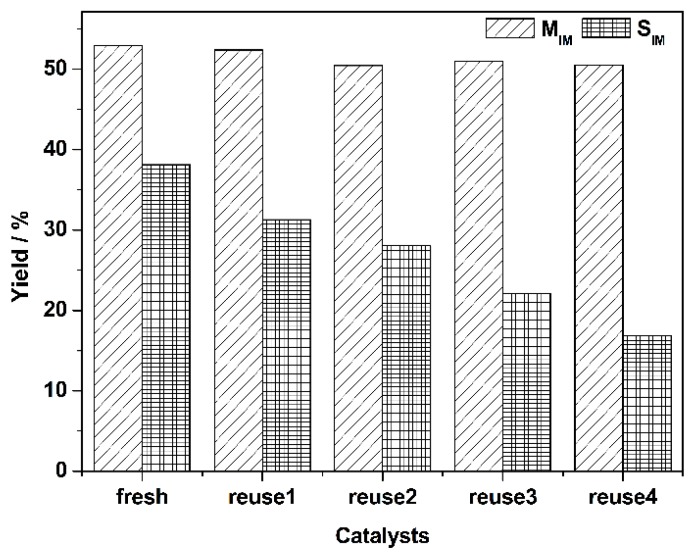
Comparison of the stabilities and reusabilities of 1% AuPd/TiO_2_ (M_Im_) and 1% AuPd/TiO_2_ (S_Im_) catalysts for the solvent free hydrogenation of NB to AN. Reaction conditions: nitrobenzene: 78 mmol, catalyst: 100 mg, temperature: 90 °C, H_2_ pressure: 3 bar, time: 6 h.

One of the crucial requirements of a heterogeneous catalyst to be used for industrial applications is its stability and reusability. Keeping that in mind, we studied the reusability of 1% AuPd/TiO_2_ (M_Im_) and 1% AuPd/TiO_2_ (S_Im_) catalysts (Figure 2). For the 1% AuPd/TiO_2_ (S_Im_) catalyst, the yield of aniline decreased from 38% to 17% after four cycles. However, for the 1% AuPd/TiO_2_ (M_Im_) catalyst, the aniline yield changed only slightly from 53% to 51%, indicating that the M_Im_ catalyst is more stable and reusable than the S_Im_ catalyst. A similar observation was previously reported by our group for the solvent-free selective oxidation of benzyl alcohol to benzaldehyde [35]. During that study, we observed that the PVA, used in the synthesis of 1% AuPd/TiO_2_ (S_Im_), blocked the active sites of the catalyst. We believe a similar phenomenon might be present hindering the reusability of the 1% AuPd/TiO_2_ (S_Im_) catalyst. On the contrary, the 1% AuPd/TiO_2_ (M_Im_) does not contain any stabilizing ligands; hence, this catalyst is more stable and reusable.

For the kinetic studies, ethanol was used as a solvent to determine the concentrations of substrate and product accurately. For all experiments, the conversion of nitrobenzene was kept to below 20% so that the time dependency of nitrobenzene concentration was linear, and to eliminate the role of products in the rate of the reaction. To ensure that the data was obtained under the kinetically controlled region, we performed the reactions at different agitation speeds, and selected an appropriate parameter (result not presented) to eliminate the effect of the mass transfer. The initial rate of the reaction was calculated at 40, 50, and 60 °C and the results are presented in Figure S1. The apparent activation energy (*Ea*) was calculated to be 37 kJ mol^−1^ from the dependence of ln(*k*) on 1/*T* with R^2^ > 0.99 (Figure S1). This value was similar to the values reported recently (37 kJ mol^−1^ over Pt/γ-Al_2_O_3_ by Peureux et al. [36], and 28 ± 5, 33 ± 5 and 45 ± 5 kJ mol^−1^ over Ru/FeO*_x_* by Easterday et al. [15], and 35 ± 1 kJ mol^−1^ over Pd/C by Turáková et al*.* [7]) for this reaction. This activation energy value also gives evidence that the reaction was carried out under a kinetic regime, otherwise the value should be in the range of 5–15 kJ mol^−1^ if the diffusion played an important role [7].

Chemoselective hydrogenation of 4-chloronitrobenzene.

The most active and stable catalyst (1% AuPd/TiO_2_ (M_Im_)) was tested for the chemoselective hydrogenation of chloronitrobenzene (CNB) to chloroaniline (CAN), because CAN is industrially very important for the synthesis of dyes, agricultural chemicals, pharmaceuticals, and polymers [37]. This reaction is also challenging because under hydrogenation conditions, typically hydrodechlorination occurs, leading to the formation of AN, hence lower CAN yield. The 1% AuPd/TiO_2_ catalysts prepared by different synthesis strategies (M_Im_, S_Im_, and C_Im_) were initially tested for the hydrogenation of CNB.

**Figure 3:**
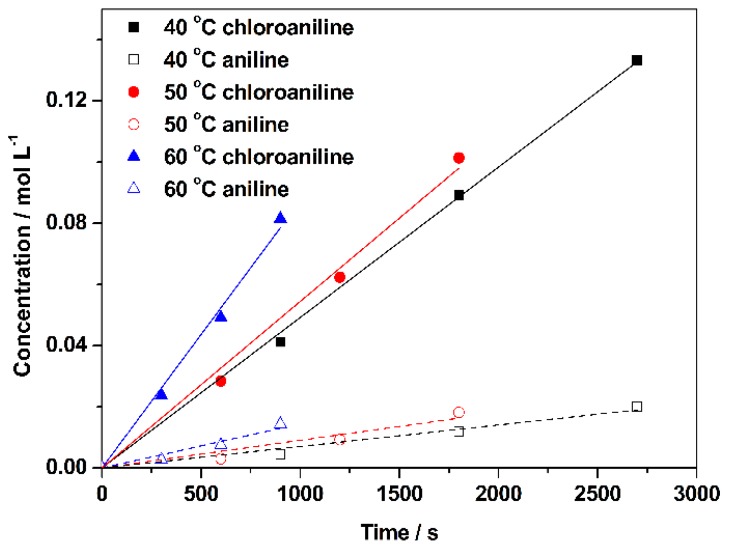
Rate of formation of 4-chloroaniline (CAN) and aniline (AN) during the hydrogenation of 4-chloronitrobenzene (CNB) over a 1% AuPd/TiO_2_ (M_Im_) catalyst at different temperatures. Reaction conditions: 4-chloronitrobenzene: 10 mmol, ethanol: 16 mL, catalyst: 12.5 mg, H_2_ pressure: 3 bar.

As shown in Table 3 Entries 1–3, the 1% AuPd/TiO_2_ (M_Im_) catalyst gave the best yield (85%) of CAN among all the catalysts tested. Using this catalyst, we then optimized the reaction conditions to increase the CAN yield. As shown in Figure 3, we performed the reactions at different temperatures at the initial stages (CNB conversion < 20%) and calculated the formation rates of 4-chloroaniline and aniline. Using these data, we calculated the apparent activation energies for CNB to CAN, and CNB to AN reactions independently, using the Arrhenius plot (Figure S2). The activation energy of CNB to CAN is 25 kJ mol^−1^, lower than that of CNB to AN (31 kJ mol^−1^). This result suggests that lower reaction temperature would be beneficial for enhancing the selectivity of CAN. Based on this data, we performed the hydrogenation of CNB at a much lower temperature (25 °C) and after 8 h of reaction time, a CAN yield of 92% was achieved (Table 3, Entry 4). This is one of the highest yields of CAN that are reported in the literature.

**Figure 4:**
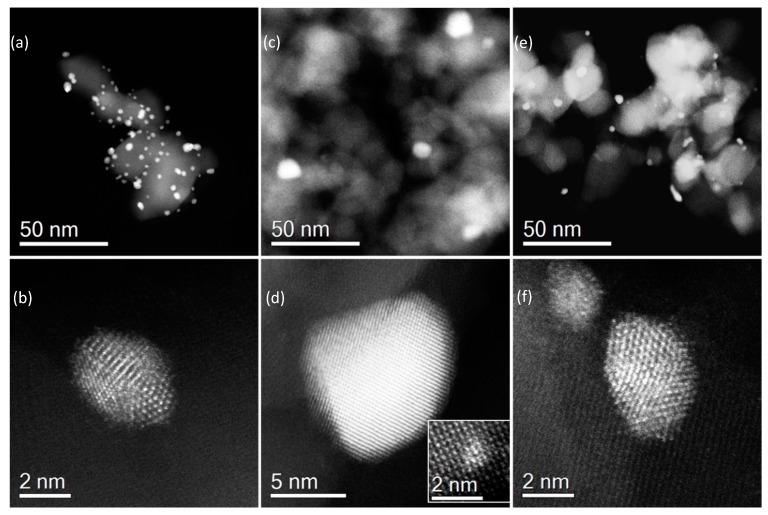
Representative HAADF-STEM images of a 1% AuPd/TiO_2_ catalyst prepared by (**a**,**b**) sol-immobilization; (**c**,**d**) conventional impregnation; (**e**,**f**) modified impregnation.

Figure 4 shows representative high angle annular dark field scanning transmission electron microscope (HAADF-STEM) images of 1% AuPd/TiO_2_ catalysts prepared by (a, b) S_Im_, (c, d) C_Im_, and (e, f) M_Im_, respectively. From the lower magnification images in Figure 4a,c,e, the C_Im_ resulted in particles of 10–20 nm in size, while both the S_Im_ and M_Im_ produced particles largely within the 2–5 nm range. From the higher-magnification HAADF images of individual particles, it can be seen that 10–20 nm particles produced by C_Im_ showed clear signs of a Pd-rich shell structure (Figure 4d). The particles produced by S_Im_ (Figure 4b) and M_Im_ (Figure 4f) appeared to be random alloys without a distinctive core-shell morphology. Some 1 nm or sub-nm clusters could also be occasionally found in the catalyst that was prepared by the C_Im_ method (inlet of Figure 4d). These were Pd-rich clusters that were probably strongly bonded to the oxide support and therefore did not merge to form bigger particles.

The electronic and geometric structures of the 1% AuPd/TiO_2_ (M_IM_) has been studied in detail in our previous papers [20,23,31]. Combining the microscopy results here and previous findings, we conclude that the higher catalytic activity and stability of 1% AuPd/TiO_2_ (M_Im_) catalyst for the solvent-free hydrogenation of NB to AN is attributed to the following reasons. In the 1% AuPd/TiO_2_ (M_Im_) catalyst, Au and Pd are efficiently mixed to form a random alloy structure instead of a traditional core–shell structure. XPS results suggested that the electron was transferred from Pd to Au, resulting in the strong electronic modification of Pd, which was considered to be one of the reasons for the enhanced catalytic activity over bimetallic catalyst compared to the monometallic Pd catalyst. In addition, the incorporation of Au atoms in the Pd lattice would result in tensile strain in the alloy structure, which caused an upward shift of the d-band of Pd, enhancing the reactivity of the surface atoms. We attribute these reasons to the higher catalytic activity of bimetallic 1% AuPd/TiO_2_ (M_Im_) catalyst than its monometallic counterparts.

Regarding the synthetic strategies, the C_IM_ resulted in a metal larger particle size, as observed in Figure 4, and as a consequence, the catalyst give a lower activity compared to the M_IM_ catalyst that gave a much smaller particle size. It should be noted that the S_IM_ methodology also resulted in smaller particle sizes similar to the M_IM_ catalyst. However in the S_Im_ catalyst, PVA was used as a stabilizing ligand, and this could block the active sites, resulting in a reduced catalytic activity for the S_Im_ catalyst. It must be noted that no stabilizing ligands were used in the M_IM_ method. Moreover, the M_IM_ underwent high-temperature reduction in the preparation procedure, which made the bimetallic Au–Pd interact strongly with the TiO_2_ support. Therefore the M_IM_ showed a better stability and reusability. The S_IM_, however, was only dried before use, resulting in a relatively less strong interaction between the bimetallic AuPd particles and the TiO_2_ support. This could be the reason for the observed deactivation behavior for the S_Im_ catalyst. 

## 4. Conclusions

In summary, we have successfully developed a 1% AuPd/TiO_2_ (M_Im_) catalyst for the solvent-free hydrogenation of nitrobenzene to aniline, and the chemoselective hydrogenation of 4-chloronitrobenzene to 4-aniline. Some important conclusions that we report in this article are as follows:

(1) Through systematic catalyst screening, 1% AuPd/TiO_2_ catalyst prepared by modified impregnation method is the most active and stable catalyst for the solvent-free hydrogenation of nitrobenzene to aniline. After a 15 h reaction, a 99% yield of aniline was obtained at 90 °C. The activation energy of nitrobenzene reduction to aniline was calculated to be 37 kJ mol^−1^.
(2) The 1% AuPd/TiO_2_ (M_Im_) catalyst also showed a good selectivity for the chemoselective hydrogenation of 4-chloronitrobenzene to 4-chloroaniline. Using the kinetic studies, we found that the activation energy of CNB transformation to CAN was calculated to be 25 kJ mol^−1^, which was lower than that of CNB to AN (31 kJ mol^−1^). Thus, by decreasing the reaction temperature from 60 °C to 25 °C, a 92% yield of CAN was achieved.

## Figures and Tables

**Table 1:** Effect of reaction temperature for the solvent-free hydrogenation of nitrobenzene over a 1% AuPd/TiO_2_ (M_Im_) catalyst ^a^.

Catalysts	Reaction Temperature (°C)	Nitrobenzene (NB) Conversion (%)	AN Selectivity (%)
1% AuPd/TiO_2_ (M_Im_)	30	1	>99
	60	11	97
	90	24	98

^a^ Reaction conditions: nitrobenzene: 78 mmol, catalyst: 100 mg, H_2_ pressure: 3 bar, time: 2 h.

**Table 2:** Effect of the different metals, supports, and synthetic strategies for the solvent-free hydrogenation of NB ^a^.

Entry	Catalysts	Conversion (%)	Selectivity (%)		
			Aniline	Azobenzene	Azoxybenzene
1	AuPd/TiO_2_ (M_Im_)	54	98	0.5	0.9
2	Au/TiO_2_ (M_Im_)	3	98	0	0
3	Pd/TiO_2_ (M_Im_)	41	97	0.2	0
4	AuPd/MgO (M_Im_)	36	94	0	0.5
5	AuPd/C (M_Im_)	14	76	0	0
6	AuPd/TiO_2_ (C_Im_)	39	99	0.4	0.3
7	AuPd/TiO_2_ (S_Im_)	38	99	0	0

^a^ Reaction conditions: nitrobenzene: 78 mmol, catalyst: 100 mg, reaction temperature: 90 °C, H_2_ pressure: 3 bar, reaction time: 6 h.

**Table 3:** Selective hydrogenation of 4-chloronitrobenzene over a AuPd/TiO_2_ catalyst ^a^.

Entry	Catalysts	Reaction Time (h)	Conversion (%)	Selectivity (%)	
				CAN	AN
1	AuPd/TiO_2_ (C_Im_)	1	96	83	17
2	AuPd/TiO_2_ (S_Im_)	1	100	70	30
3	AuPd/TiO_2_ (M_Im_)	1.5	100	85	15
4	AuPd/TiO_2_ (M_Im_) ^b^	8	100	92	8

^a^ Reaction conditions: 4-chloronitrobenzene: 10 mmol, ethanol: 16 mL, catalyst: 12.5 mg, temperature: 60 °C, H_2_ pressure: 3 bar. ^b^ Reaction temperature 25 °C.

## Supplementary Materials

The following are available online at http://www.mdpi.com/2079-4991/8/9/690/s1, Figure S1: Arrhenius plot for the hydrogenation of nitrobenzene, Figure S2: Arrhenius plot for the hydrogenation of CAN.

## References

[1] Wang J.H., Yuan Z.L., Nie R.F., Hou Z.Y., Zheng X.M. (2010). Hydrogenation of nitrobenzene to aniline over silica gel supported nickel catalysts. Ind. Eng. Chem. Res..

[2] Lara P., Philippot K. (2014). The hydrogenation of nitroarenes mediated by platinum nanoparticles: An overview. Catal. Sci. Technol..

[3] Wei H.S., Liu X.Y., Wang A.Q., Zhang L.L., Qiao B.T., Yang X.F., Huang Y.Q., Miao S., Liu J.Y., Zhang T. (2014). FeO*_x_*-supported platinum single-atom and pseudo-single-atom catalysts for chemoselective hydrogenation of functionalized nitroarenes. Nat. Commun..

[4] Sun Z.Y., Zhao Y.F., Xie Y., Tao R.T., Zhang H.Y., Huang C.L., Liu Z.M. (2010). The solvent-free selective hydrogenation of nitrobenzene to aniline: An unexpected catalytic activity of ultrafine Pt nanoparticles deposited on carbon nanotubes. Green Chem..

[5] Rylander P.N. (1990). Hydrogenation Methods.

[6] Nishimura S. (2001). Handbook of Heterogeneous Catalytic Hydrogenation for Organic Synthesis.

[7] Turáková M., Salmi T., Eränen K., Wärnå J., Murzin D.Y., Králik M. (2015). Liquid phase hydrogenation of nitrobenzene. Appl. Catal. A.

[8] Hu Y.B., Tao K., Wu C.Z., Zhou C., Yin H.F., Zhou S.H. (2013). Size-controlled synthesis of highly stable and active Pd@SiO_2_ core-shell nanocatalysts for hydrogenation of nitrobenzene. J. Phys. Chem. C.

[9] Torres C., Campos C., Fierro J., Oportus M., Reyes P. (2013). Nitrobenzene hydrogenation on Au/TiO_2_ and Au/SiO_2_ catalyst: Synthesis, characterization and catalytic activity. Catal. Lett..

[10] Du W.C., Chen G.Z., Nie R.F., Li Y.W., Hou Z.Y. (2013). Highly dispersed Pt in MIL-101: An efficient catalyst for the hydrogenation of nitroarenes. Catal. Commun..

[11] Raj K.J.A., Prakash M.G., Mahalakshmy R., Elangovan T., Viswanathan B. (2012). Liquid phase hydrogenation of nitrobenzene over nickel supported on Titania. Chin. J. Catal..

[12] Manzoli M., Shetti V.N., Blaine J.A.L., Zhu L., Isrow D., Yempally V., Captain B., Coluccia S., Raja R., Gianotti E. (2012). Ru*_x_*Pt*_y_*Sn*_z_* cluster-derived nanoparticle catalysts: Spectroscopic investigation into the nature of active multinuclear single sites. Dalton Trans..

[13] Huang X.Q., Li Y.J., Li Y.J., Zhou H.L., Duan X.F., Huang Y. (2012). Synthesis of PtPd bimetal nanocrystals with controllable shape, composition, and their tunable catalytic properties. Nano Lett..

[14] Turáková M., Králik M., Lehocký P., Pikna L., Smrčová M., Remeteiová D., Hudák A. (2014). Influence of preparation method and palladium content on Pd/C catalysts activity in the liquid phase hydrogenation of nitrobenzene to aniline. Appl. Catal. A.

[15] Easterday R., Sanchez-Felix O., Losovyj Y., Pink M., Stein B.D., Morgan D.G., Rakitin M., Doluda V.Y., Sulman M.G., Mahmoud W.E. (2015). Design of ruthenium/iron oxide nanoparticle mixtures for hydrogenation of nitrobenzene. Catal. Sci. Technol..

[16] Sheldon R.A. (2017). The *E* factor 25 years on: The rise of green chemistry and sustainability. Green Chem..

[17] Huang H., Wang X., Tan M., Chen C., Zou X., Ding W., Lu X. (2016). Solvent-free selective hydrogenation of nitroarenes using nanoclusters of palladium supported on nitrogen-doped ordered mesoporous carbon. ChemCatChem.

[18] Bianchi C.L., Canton P., Dimitratos N., Porta F., Prati L. (2005). Selective oxidation of glycerol with oxygen using mono and bimetallic catalysts based on Au, Pd and Pt metals. Catal. Today.

[19] Enache D.I., Edwards J.K., Landon P., Solsona-Espriu B., Carley A.F., Herzing A.A., Watanabe M., Kiely C.J., Knight D.W., Hutchings G.J. (2006). Solvent-free oxidation of primary alcohols to aldehydes using AuPd/TiO_2_ catalysts. Science.

[20] Luo W., Sankar M., Beale A.M., He Q., Kiely C.J., Bruijnincx P.C.A., Weckhuysen B.M. (2015). High performing and stable supported nano-alloys for the catalytic hydrogenation of levulinic acid to γ-valerolactone. Nat. Commun..

[21] Freakley S.J., He Q., Harrhy J.H., Lu L., Crole D.A., Morgan D.J., Ntainjua E.N., Edwards J.K., Carley A.F., Borisevich A.Y. (2016). Palladium-tin catalysts for the direct synthesis of H_2_O_2_ with high selectivity. Science.

[22] Agarwal N., Freakley S.J., McVicker R.U., Althahban S.M., Dimitratos N., He Q., Morgan D.J., Jenkins R.L., Willock D.J., Taylor S.H. (2017). Aqueous Au-Pd colloids catalyze selective CH_4_ oxidation to CH_3_OH with O_2_ under mild conditions. Science.

[23] Sankar M., Dimitratos N., Miedziak P.J., Wells P.P., Kiely C.J., Hutchings G.J. (2012). Designing bimetallic catalysts for a green and sustainable future. Chem. Soc. Rev..

[24] Hutchings G.J., Kiely C.J. (2013). Strategies for the synthesis of supported gold palladium nanoparticles with controlled morphology and composition. Acc. Chem. Res..

[25] Tafesh A.M., Weiguny J. (1996). A review of the selective catalytic reduction of aromatic nitro compounds into aromatic amines, isocyanates, carbamates, and ureas using CO. Chem. Rev..

[26] Iihama S., Furukawa S., Komatsu T. (2016). Efficient catalytic system for chemoselective hydrogenation of halonitrobenzene to haloaniline using PtZn intermetallic compound. ACS Catal..

[27] Lyu J.H., Wang J.G., Lu C.S., Ma L., Zhang Q.F., He X.B., Li X.N. (2014). Size-dependent halogenated nitrobenzene hydrogenation selectivity of Pd nanoparticles. J. Phys. Chem. C.

[28] Wei Q., Shi Y.S., Sun K.Q., Xu B.Q. (2016). Pd-on-Si catalysts prepared via galvanic displacement for the selective hydrogenation of para-chloronitrobenzene. Chem. Commun..

[29] Zhang J., Wang Y., Ji H., Wei Y., Wu N., Zuo B., Wang Q. (2005). Magnetic nanocomposite catalysts with high activity and selectivity for selective hydrogenation of ortho-chloronitrobenzene. J. Catal..

[30] Cárdenas-Lizana F., Gómez-Quero S., Keane M.A. (2008). Clean production of chloroanilines by selective gas phase hydrogenation over supported Ni catalysts. Appl. Catal. A.

[31] Sankar M., He Q., Morad M., Pritchard J., Freakley S.J., Edwards J.K., Taylor S.H., Morgan D.J., Carley A.F., Knight D.W. (2012). Synthesis of stable ligand-free gold–palladium nanoparticles using a simple excess anion method. ACS Nano.

[32] Peneau V., He Q., Shaw G., Kondrat S.A., Davies T.E., Miedziak P., Forde M., Dimitratos N., Kiely C.J., Hutchings G.J. (2013). Selective catalytic oxidation using supported gold-platinum and palladium-platinum nanoalloys prepared by sol-immobilisation. Phys. Chem. Chem. Phys..

[33] Kesavan L., Tiruvalam R., Ab Rahim M.H., bin Saiman M.I., Enache D.I., Jenkins R.L., Dimitratos N., Lopez-Sanchez J.A., Taylor S.H., Knight D.W. (2011). Solvent-free oxidation of primary carbon-hydrogen bonds in toluene using Au-Pd alloy nanoparticles. Science.

[34] Landon P., Collier P.J., Carley A.F., Chadwick D., Papworth A.J., Burrows A., Kiely C.J., Hutchings G.J. (2003). Direct synthesis of hydrogen peroxide from H_2_ and O_2_ using Pd and Au catalysts. Phys. Chem. Chem. Phys..

[35] Morad M., Sankar M., Cao E.H., Nowicka E., Davies T.E., Miedziak P.J., Morgan D.J., Knight D.W., Bethell D., Gavriilidis A. (2014). Solvent-free aerobic oxidation of alcohols using supported gold palladium nanoalloys prepared by a modified impregnation method. Catal. Sci. Technol..

[36] Peureux J., Torres M., Mozzanega H., Giroir-Fendler A., Dalmon J.A. (1995). Nitrobenzene liquid-phase hydrogenation in a membrane reactor. Catal. Today.

[37] Serpell C.J., Cookson J., Ozkaya D., Beer P.D. (2011). Core@shell bimetallic nanoparticle synthesis via anion coordination. Nat. Chem..

