# Supplementary material for: Supported Bimetallic AuPd Nanoparticles as a Catalyst for the Selective Hydrogenation of Nitroarenes
Source: Nanomaterials (Basel). 2018 Sep 5;8(9):690. doi: 10.3390/nano8090690 (PMC6165381; doi:10.3390/nano8090690)
Supplement: Supplementary file 1 [file nanomaterials-08-00690-s001.pdf]

Supporting information

Supported Bimetallic AuPd Nanoparticles as a Catalyst for the Selective Hydrogenation of Nitroarenes

Ruiyang Qu, Margherita Macino, Sarwat Iqbal, Xiang Gao*, Qian He, Graham John Hutchings and Meenakshisundaram Sankar*

[a] *Cardiff Catalysis Institute, School of Chemistry, Cardiff University, Cardiff, CF10 3AT, UK*

[b] *State Key Laboratory of Clean Energy Utilization, College of Energy Engineering, Zhejiang University, Hangzhou, 310027 ,China*

* *Corresponding author. Prof. X. Gao, State Key Laboratory of Clean Energy Utilization, College of Energy Engineering, Zhejiang University, Hangzhou, 31002, China.*

E-mail: xgao1@zju.edu.cn

* *Corresponding author. Dr. M. Sankar, Cardiff Catalysis Institute, School of Chemistry, Cardiff University, Cardiff, CF10 3AT, UK*

E-mail address: Sankar@cardiff.ac.uk

This supporting information contains

Figures S1and S2

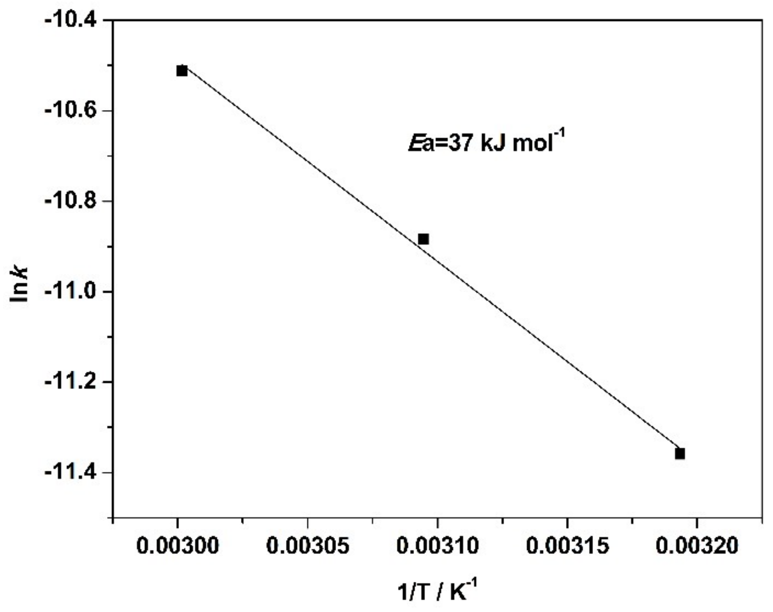

Figure S1 *Arrhenius plot for the hydrogenation of nitrobenzene over 1%AuPd/TiO_2 (M_{Im}) catalyst. Reaction conditions: nitrobenzene: 10 mmol, ethanol: 16 mL, catalyst: 12.5 mg, H_2 pressure: 3 bar.*

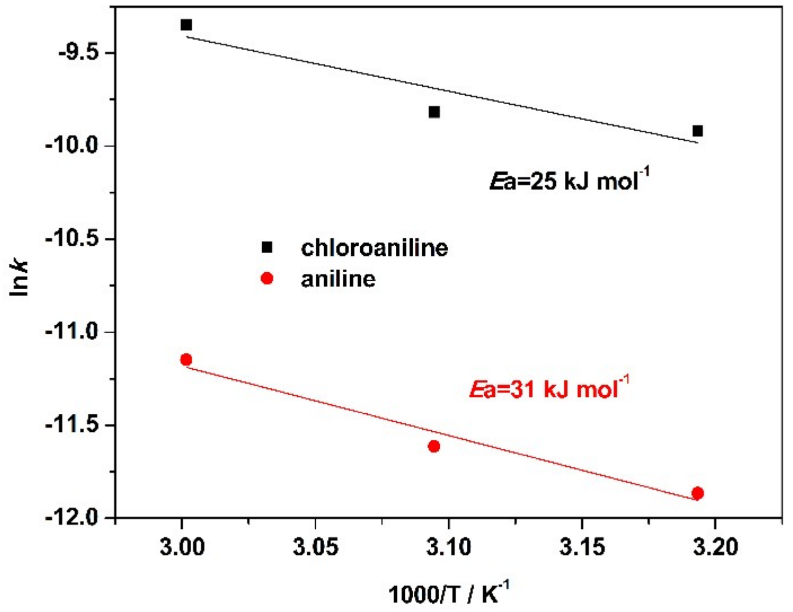

Figure S2 *Arrhenius plot for the hydrogenation of CAN over 1%AuPd/TiO_2 (M_{Im}) catalyst. Reaction conditions: CAN: 10 mmol, ethanol: 16 mL, catalyst: 12.5 mg, H_2 pressure: 3 bar.*
